# The subsurface urban heat island of Basel City: more than a decade of spatiotemporal high-resolution monitoring and modelling

**DOI:** 10.1098/rsta.2024.0577

**Published:** 2025-11-06

**Authors:** Martin Binder, Falk Händel, Christian Engelmann, Brian Steiner, Alma Johanna Hasler, Alejandro García Gil, Jannis Epting

**Affiliations:** ^1^ Hydrogeology / Applied and Environmental Geology – AUG, University of Basel, Basel 4056, Switzerland; ^2^ Institute of Geology / Chair of Hydrogeology and Hydrochemistry, Technische Universität Bergakademie Freiberg, Freiberg 09599, Germany; ^3^ Institute of Geotechnics / Chair of Soil Mechanics and Foundation Engineering, Technische Universität Bergakademie Freiberg, Freiberg 09599, Germany; ^4^ Department of Monitoring and Exploration Technologies, Helmholtz-Centre for Environmental Research – UFZ, Leipzig 04318, Germany; ^5^ Geological and Mining Institute of Spain – IGME, Spanish National Research Council – CSIC, Madrid 28003, Spain

**Keywords:** subsurface urban heat island (SUHI), depth-oriented temperature monitoring, thermo-hydraulic (TH) model

## Abstract

Managing urban groundwater resources is crucial not only for water quantity questions, but also to safeguard water quality. While evaluating hydrochemical parameters is part of common monitoring strategies, given the ongoing trend of geothermal energy usage, the evaluation of thermal regimes has gained increasing interest. This study presents an analysis of groundwater temperature (GWT) datasets from conventional monitoring networks and from seven high-resolution multi-level monitoring systems in Basel City, Switzerland. With GWT hotspots of up to 20°C, the monitoring data clearly showed the transient development of a subsurface urban heat island (SUHI). An existing suite of three-dimensional thermal hydraulic (3D-TH) models for four distinct groundwater bodies (GWB) was updated to enable SUHI analysis. For this, GWT, groundwater heads, river temperatures, river stages and groundwater user data were merged and introduced into the 3D-TH, enabling a 6-year calibration plus a 5-year validation period. The updated models provide insights into the long-term groundwater and heat flux dynamics across Basel’s GWB. The findings underscore the flexibility of monitoring and modelling in evaluating urban groundwater systems, promoting a sustainable use and management of shallow geothermal energy and formulating SUHI mitigation strategies.

This article is part of the theme issue ‘Urban heat spreading above and below ground’.

## 1. Introduction

Anthropogenic activities have increased shallow subsurface and groundwater temperatures (GWT) in urban areas compared to natural thermal states [1, and references therein]. Urban GWT are jointly influenced by natural and anthropogenic factors [2–4], including climatic impacts, river-groundwater interaction, infrastructure (e.g. district heating, sewage networks, heated buildings, tunnels, surface sealing, underground car parks [5,6]) and open/closed-loop geothermal systems. Competing subsurface uses further impact urban GWT regimes on local and city scales [7,8]. Elevated urban GWT compared to natural thermal states [2,9] are observed in numerous regions worldwide and termed as the subsurface urban heat island (SUHI) effect. The long-term and large-scale warming of the subsurface is especially critical: it accelerates geochemical reactions [10], alters microbial communities [11,12] as well as groundwater ecosystems in general [13,14], and can also reduce the efficiency of shallow-geothermal systems (especially cooling), making a reliable characterization of SUHI essential for sustainable groundwater management. Detecting and managing SUHI requires multi-decadal, high-resolution monitoring capable of distinguishing natural climatic variability from anthropogenic heat fluxes and of providing a sound basis for regulatory action.

In Basel City (Switzerland), as presented in this case study, GWT have been monitored for almost four decades; first manually for a few selected sites, later city-wide by using digital sensors in conventional observation wells (OW) as well as multi-level ground monitoring systems (GMS). The SUHI effect in Basel City is clearly visible, with annual GWT often exceeding natural thermal states (10–11°C) due to anthropogenic factors [1], with extremes of up to approx. 20°C observed by the cantonal authorities (Agency for Environment and Energy Basel City (AUE BS) and Agency for Environmental Protection and Energy Basel-Landscape (AUE BL)). The Basel GWT archive is among the longest and most densely sampled SUHI data sets worldwide, on a par with, e.g. the approx. 30-year record that underpins the well-known SUHI assessment of Cologne (Germany). Several European cities, notably Zaragoza (Spain), Ljubljana (Slovenia), Munich (Germany) and Cardiff (United Kingdom), have recently begun to digitize their GWT networks (since 2010; discussed in [15]) and may benefit from the recent Basel experiences. Going beyond the classical monitoring of thermal (GWT) and hydraulic (groundwater head, GWH) process variables, previous studies [1,3,16,17] in Basel City actively combined monitoring with numerical three-dimensional thermal-hydraulic (3D-TH) models, while following the groundwater body (GWB) approach on a city district scale. Due to previous limitations in digital data availability, the existing numerical model (FEFLOW^©^, DHI Group [18]) used a short 1 year calibration period (year 2010; model version of [17]).

This study aims to give a brief overview of recent developments in Basel City regarding SUHI monitoring and modelling. With a special focus on the last decade, we (i) quantify thermal trends in the four GWB of Basel City, (ii) characterize vertical heat transfer processes and preferential heat transport through high-permeable gravels by evaluating seven GMS profiles in the unsaturated and saturated zones, and (iii) update, calibrate and validate the 3D-TH models for each GWB, providing a robust tool for future SUHI forecasts and management.

## 2. Description of the study site and the data inventory

(a) Location, geology and hydrogeology

**Figure 1.**
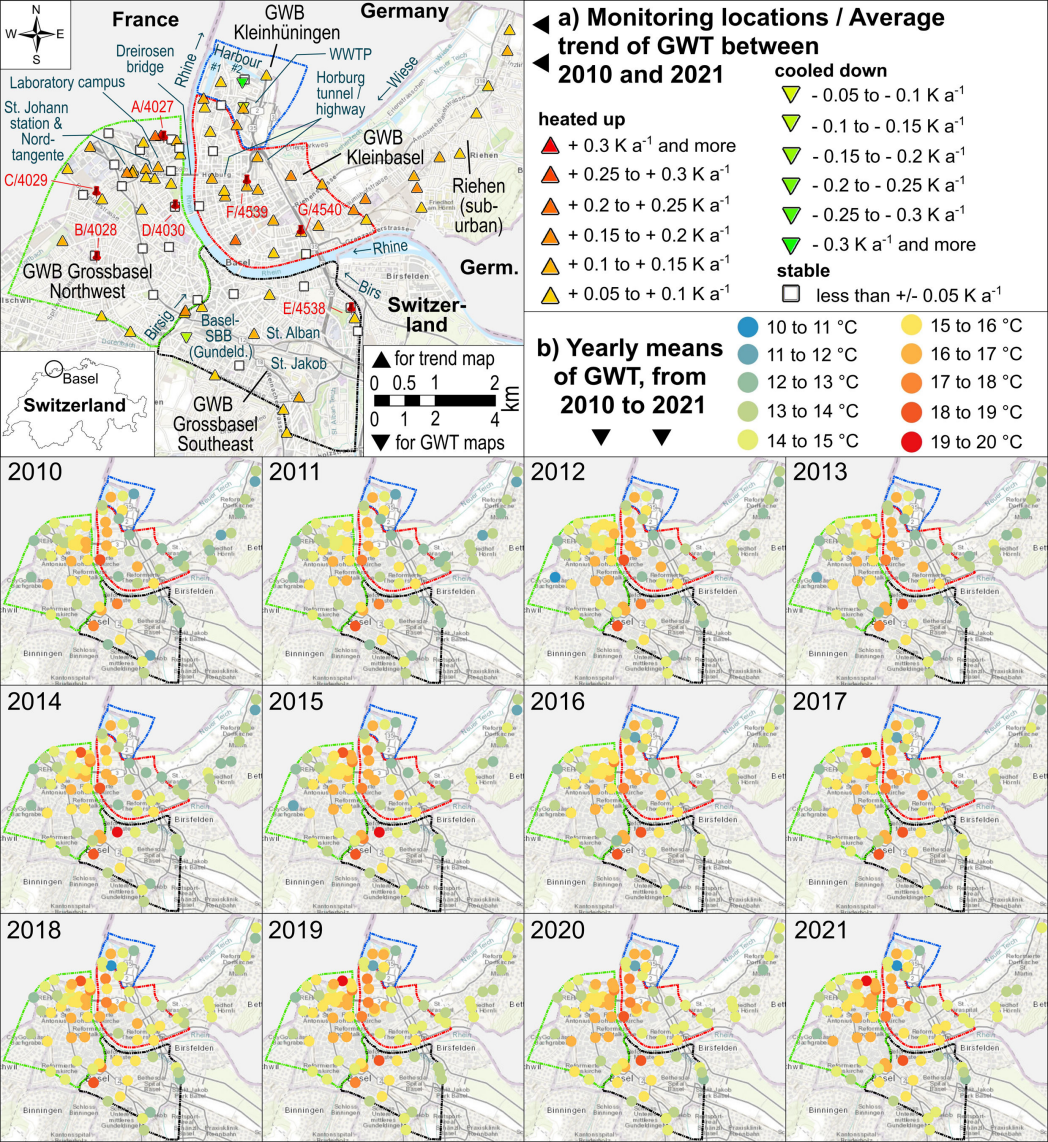
Study area of the Swiss canton Basel City including the location of seven multi-level temperature GMS (a; labelled ‘A’ to ‘G’) and relevant constructions (deep buildings and sewer network in electronic supplementary material, figures S22 and S23, respectively; wastewater treatment plant is labelled ‘WWTP’). Furthermore, the trend of GWT as observed in conventional groundwater OW (a) and the respective means of selected years are displayed (b). The four areas with the dashed borders denote the four selected GWB used for the 3D-TH modelling, namely ‘Grossbasel Northwest’ (green area); ‘Grossbasel Southeast’ (black); ‘Kleinbasel’ (red) and ‘Kleinhüningen’ (blue). The suburban region Riehen is located northeast of Kleinbasel.

The study area covers the canton of Basel City (approx. 32 km²), Switzerland (figure 1a). Four models for the GWB ‘Grossbasel Northwest’, ‘Grossbasel Southeast’, ‘Kleinbasel’ and ‘Kleinhüningen’ were previously developed [16,17] and updated stepwise incorporating documented subsurface structures, such as underground garages and basements [19,20]. Partially, these four GWB share the same hydraulic and thermal boundaries (e.g. surface waters), acting as connecting elements between the GWB.

The near-surface gravel aquifer is up to 38 m thick, consisting mainly of unconsolidated alluvial sediments deposited by the local rivers (Rhine and its tributaries Wiese, Birs and Birsig). This highly permeable gravel aquifer is underlain by mud/clay-rich sediments acting as hydraulically irrelevant but thermally relevant aquiclude [8]. The model top was directly derived from digital elevation models (DEM; 2 × 2 m² resolution) and high-precision echo sounding of the river Rhine [21]. The transition level between the aquifer and the aquiclude is based on an existing geological model (approx. 4000 drill cores [21], employing modelling software Aspen SKUA by Aspen Technology Inc.).

**Figure 2.**
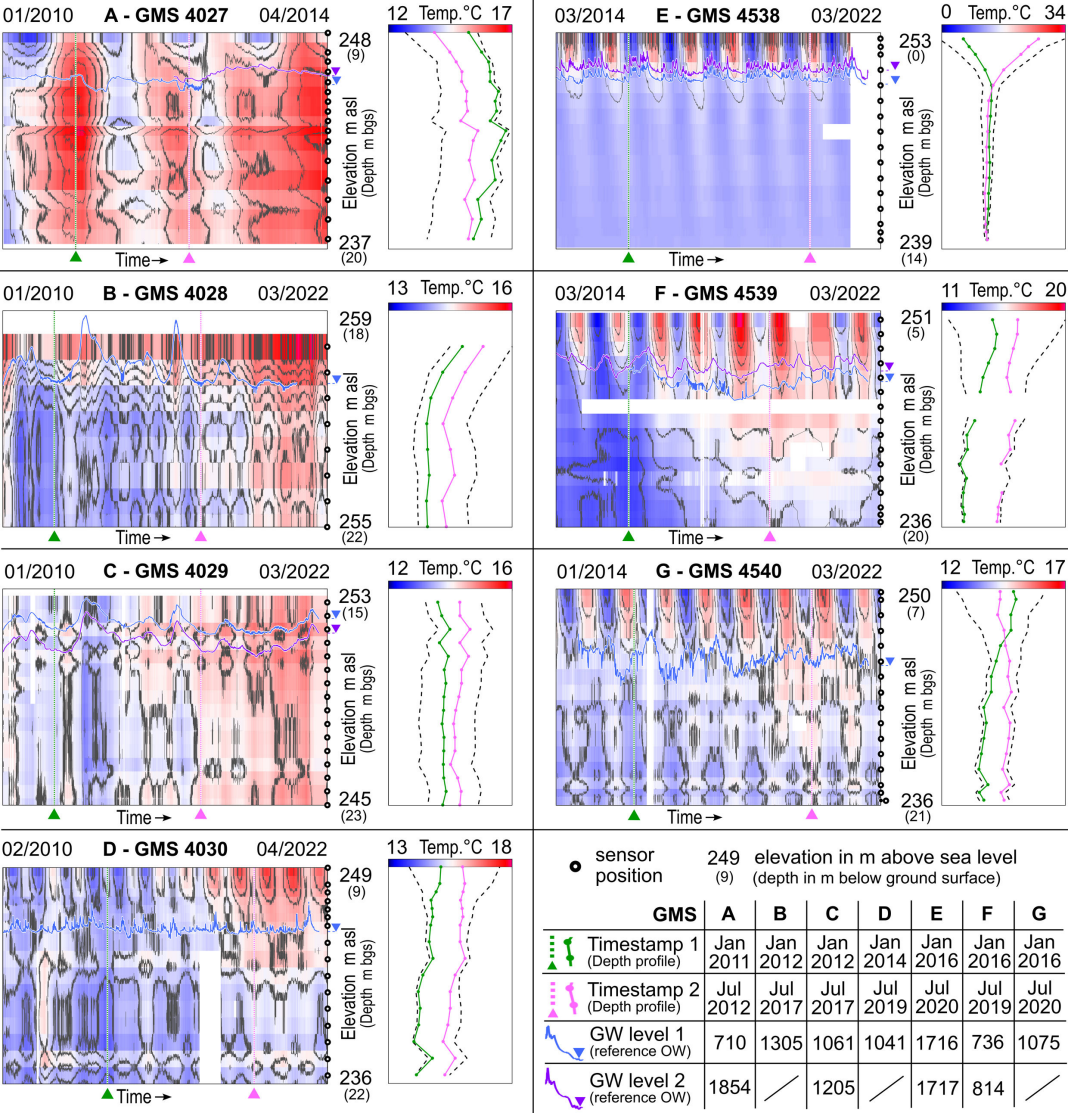
Spatiotemporal development of temperatures for the seven high-resolution multi-level GMS (A: 4027, B: 4028, C: 4029, D: 4030, E: 4538, F: 4539, and G: 4540) and hydraulics (from nearby OW). Left (in each subfigure): interpolation and temporal development of GWT and UZT data. Right: Tautochrones showing all sensor measurements with two situations highlighted. The vertical axis (both graphs) shows elevation and sensor depth bgs, respectively. Vertical green and pink lines depict selected scenarios, and blue and violet lines depict reference GWH (from nearby OW).

GWH and GWT data were contributed by the long-term monitoring programme of AUE-BS and AUE-BL. Both digital logger data as well as manually compiled data from hydrological yearbooks were used to create the observation time series. GWH vary significantly, with minima of a few metres below surface in Kleinbasel and Kleinhüningen (right side of the river Rhine; figure 1) and maxima of up to 30 m in Grossbasel (left side of the river Rhine; figure 1). The thickness of the saturated zone ranges from low single digits to 16 m during medium groundwater regime [17]. The monitoring data was complemented with data contributed by the local drinking water supplier (Industrielle Werke Basel; IWB). Furthermore, seven high-resolution, multi-level GMS, each equipped with 8 to 16 thermistor sensors at 0.5‒1 m depth intervals (Pt100; Ott Logosens; ± 0.1 K accuracy; 0.01 K resolution), were installed at selected sites (labelled ‘A’ to ‘G’ in figures 1 and 2; four systems in 2010; additionally, three systems in 2014) in Basel City to investigate vertical heat transport in the vadose zone and the thermal regime (e.g. [1,3,16,17,22]).

### (b) Impacts by surface water and atmosphere

River stages and water temperatures of the rivers Rhine and its tributaries Wiese, Birs and Birsig were obtained by the Federal Office for the Environment (FOEN), and partially by the civil engineering department of Basel-Landscape (TBA BL) and IWB. Water levels were interpolated linearly between the monitoring stations. Water levels of the harbour area in Kleinhüningen were monitored by ‘Schweizerische Rheinhäfen’, jointly with FOEN, and assumed to be spatially constant. The temperature data of the river monitoring stations was assigned to the entire river water bodies for simplification purposes, assuming a fast advective heat transport with the flowing water.

The mean annual precipitation of the Basel region is approx. 821 mm based on the period 2010 to 2021 (source: meteorological station Basel-Binningen operated by MeteoSwiss). The trend in this period is −7.2 mm a^−1^. Basel City has a temperate oceanic climate [17] with monthly air temperatures ranging from −2.2°C to 23.2°C between 2010 and 2021 (mean: 11.3°C; trend: +0.09 K a^−1^). Temperatures in the shallow subsurface (soil, 20 cm below surface; MeteoSwiss) are measurably higher than air temperatures with a mean value of 13.5°C and a trend of almost +0.15 K a^−1^ in the last decade. The long-term mean values (30 year long period since 1991; MeteoSwiss) for precipitation, air and soil temperatures are approx. 864 mm, 11.0°C and 12.4°C with trend values of −2.6 mm a^−1^,+0.04 K a^−1^ and +0.06 K a^−1^, respectively. Basel City is officially considered as an urban heat island region [17,20]; given these accelerating trends, this applies to both above and below the surface.

### (c) Anthropogenic influence by industrial-scale geothermal users and by artificial subsurface structures

Between a minimum of 74 (year 2012) and a maximum of 95 (year 2020) extraction/injection sites, partially grouped to industrial-scale users with noticeable thermal relevance, were operating in the period from 2010 to 2021. The respective water abstraction and recharge volumes are reported annually from the thermal users to the corresponding authorities AUE BS and AUE BL. The annually extracted groundwater volume in the four GWB regions for non-domestic usage (excluding the main drinking water extraction area of ‘Lange Erlen’ northeast of the city of Basel; figure 1) slightly increased from a minimum of approx. 8.0 × 10^6^ m^3^ in 2010 to 1.0 × 10^7^ m^3^ in 2021, with a maximum of 1.1 × 10^7^ m^3^ in 2020. The volume of reinjected ‘warm’ water (cooling operation mode) varied between a minimum of approx. 9.2 × 10^5^ m^3^ in 2015 and 1.7 × 10^6^ m^3^ in 2021. Contrarily, the reinjection of ‘cold’ water (heating operation mode) is much lower, with annual volumes ranging only between approx. 3.5 × 10^5^ m^3^ in 2011 and 6.2 × 10^5^ m^3^ in 2016. For more information (volumes over time, usage types), please refer to electronic supplementary material, figure S1. For a limited number of users, GWT monitoring data was available for withdrawal and/or reinjection. In these cases, the data was directly implemented in the 3D-TH models. Elsewhere, a correct working condition within a predefined temperature range, assigned on the basis of the initially requested operation type (i.e. cooling, heating, combination), was assumed.

For later use in the models, temperature time series are required for miscellaneous subsurface constructions (e.g. ordinary buildings, tunnels, underground car parks and deep buildings). Since these datasets are not available for the vast majority, a combination of air temperature data (used when air temperature is 12°C or above) and heating day degree data (when below 12°C) was used to generate these time series. Hereby, a local polynomial regression was applied to the transition points—specifically, the locally estimated scatterplot smoothing (LOESS) approach was chosen (more details in [3] and [17]). Three-dimensional geometry data related to these constructions was based on data provided by AUE BS and by the building insurance of Basel City [19,20].

## 3. SUHI-related GWT monitoring and modelling in Basel City

### (a) Conventional observation wells

Figure 1 gives a brief overview of the groundwater thermal regime based on GWT data from conventional OW (2010 to 2021) for all four GWB of Basel City, as well as the suburban Riehen area to the northeast. Shown are the average trend (in K a^−1^) and the inter-annual arithmetic means (the monthly GWT are available in digital form in the electronic supplementary material). The latter were calculated wherever complete data records were available for the respective year (i.e. 12 months of data without major data gaps).

Elevated GWT can be found in almost all regions but with a strong focus on the urbanized areas (these regions are covering between 84.7% (Kleinhüningen) and 91.3% (Grossbasel Southeast) of the total GWB areas). Already in 1998 (see annual means in electronic supplementary material, figure S3), GWT hotspots of up to 16.4°C were observed in the core regions of Kleinbasel and of Grossbasel Northwest, while the border regions were comparably cold with GWT only slightly above the natural background. Over the years, SUHI developed as location-dependent, though some thermal impact types can be found in all GWB. This includes, e.g. large-scale subsurface structures such as the sewer network (mean sewer water temperature of 18.8°C; [5]) that crisscrosses Basel City with a cumulative length of more than 600 km [5]. Please refer to the electronic supplementary material for enlarged maps including changes between the years (figures S4–S15), for GWB-sorted GWT time series (figures S16–S20), as well as for maps of thermally relevant structures such as sewer network, tunnels and buildings (figures S21 and S23).

A positive trend applies in large parts of Kleinbasel—‘positive’ in the quantitative context with detrimental GWT trends by up to 0.25 K increase per year; these extremes are very local, but the regional annual mean GWT also rose from 14.3°C to 14.9°C (figure 1a). This area is an urban area with a wide range of thermal impacts, including geothermal use for heating and cooling. Four OW in Kleinbasel show no trend: (i) two OW, 1331 and 1665, near the rivers Rhine and Wiese, respectively, seem to be more influenced by the rivers than by anthropogenic structures nearby (however, effects are overlapping here which does not enable a clear distinction between them); (ii) OW 703 near the river Rhine, which is also located near a main sewer line, shows an elevated but stable GWT; and (iii) the more central OW 813 is potentially influenced by the ‘Horburg’ tunnel and highway, also with a constantly high GWT. The eastern area of Kleinbasel, characterized by very shallow groundwater, shows a total increase of 1.4 K since 2010 (and of 2.7 K if 1998 is taken as reference year). The same applies for the adjacent suburban area of Riehen, at the end of the investigated period almost reaching absolute values like Kleinbasel (values rose from 12.9°C to 14.1°C), i.e. the ongoing spread of SUHI into that region is visible. Basel City’s northern harbour area (Kleinhüningen; averaged GWT rose from 13.7°C to 14.8°C) has OW with contradicting trends and is also heterogeneous regarding absolute GWT. In 2021, there are comparably warm regions near the wastewater treatment plant and the ‘harbour basin #1’ (next to the river Rhine), while comparably cold GWT were observed for the area nearby the northern ‘harbour basin #2’. Here, the GWT trend is negative. Contrary to the areas right of the river Rhine, large portions of the Grossbasel region left of the river Rhine, especially the central parts and the area near the river Birsig, are characterized by high GWT, many of them on a stable level (±0.05 K a^−1^). The average GWT of Grossbasel Northwest and Grossbasel Southeast in 2010 are 14.7°C and 14.3°C, respectively; 15.2°C and 14.8°C are the recorded annual mean values of 2021. This whole area is densely urbanized with noticeable GWT hotspots (e.g. more than 20°C in summer 2018). The large central train station ‘Basel-SBB’ is located there in ‘Gundeldingen’ (mainly Grossbasel Southeast), and it is directly accompanied by a large-coverage railway infrastructure in the adjacent ‘St. Alban’ and ‘St. Jakob’ area. The northern section of Grossbasel Northwest, near to the ‘Dreirosen’ bridge, has recently seen increases in GWT. The ‘St. Johann’ train station, the tunnel structure belonging to the city tunnel highway ‘Nordtangente’, as well as the laboratory campus of a pharmacy company are assumed by the authors to be some of the major reasons for this—here, a more in-detail investigation is necessary.

### (b) Location-specific effects (high-resolution multi-level monitoring)

Figure 2 gives a comparative overview of the seven high-resolution, multi-level GMS 4027 to 4030 and 4538 to 4540. Enlarged versions of the datasets can be found in the electronic supplementary material, figures S24 (map of locations) and S26–S31. These GMS are based on temperature sensors directly embedded in the rock matrix of the vadose/unsaturated zone (USZ) and the groundwater-saturated zone (SZ). Table 1 shows sensor installation data, minimum and maximum temperatures, as well as trends, in USZ and SZ in comparison to data measured in nearby conventional OW.

**Table 1.** Statistics including trends of temperatures measured in GMS (multiple sensors per observation) and OW nearby (one sensor per observation). The OW nearby are listed below the respective GMS.

	sensor numbe**r**	sensor depth range	sensor in SZ (at depth)	temperatures in SZ		trend in SZ	sensor in USZ (at depth)	temperatures in USZ		trend in USZ
		[m bgs]	S# [m bgs]	T_Min_ [°C]	T_Max_ [°C]	[K a^−1^]	S# [m bgs]	T_Min_ [°C]	T_Max_ [°C]	[K a^−1^]
**GMS 4027**	16x	9.0–19.5	S4 (16.5)	14.1	16.6	2.1 × 10^–1^	S15 (10.0)	13.5	15.9	2.4 × 10^−1^
OW 710	—	—	—	14.8	16.3	−1.8 × 10^–1^	—	—	—	—
OW 1854	—	—	—	13.6	20.8	3.5 × 10^–1^	—	—	—	—
**GMS 4028**	8x	18.5–22.0	S2 (21.5)	13.7	14.9	5.9 × 10^−2^	S8 (18.5)	14.5	16	7.7 × 10^−2^
OW 1305	—	—	—	14.7	17.4	4.4 × 10^−2^	—	—	—	—
**GMS 4029**	16x	15.5–23.0	S4 (21.5)	13	14.7	5.7 × 10^−2^	S16 (15.5)	13.2	15	9.7 × 10^−2^
OW 1061	—	—	—	13.7	14.3	3.5 × 10^−2^	—	—	—	—
OW 1205	—	—	—	14.7	15.6	5.3 × 10^−2^	—	—	—	—
**GMS 4030**	16x	9.0–21.5	S3 (19.5)	14	15.6	4.1 × 10^−2^	S15 (10.0)	14.2	17.2	1.8 × 10^−1^
OW 1041	—	—		15.1	16.3	4.4 × 10^−2^		—	—	—
**GMS 4538**	16x	0.4–13.4	S4 (11.4)	11.5	13.3	2.3 × 10^−2^	S15 (0.9)	2.3	28.6	−1.7 × 10^−1^
OW 1716	—	—		11.5	14.7	4.4 × 10^−3^	—	—	—	—
OW 1717	—	—		9.9	16.0	4.4 × 10^−2^	—	—	—	—
**GMS 4539**	16x	5.6–19.6	S5 (16.6)	12.7	16	3.7 × 10^−1^	S15 (6.6)	12.2	19.4	2.9 × 10^−1^
OW 736	—	—	—	14.5	18.0	2.6 × 10^−1^	—	—	—	—
OW 814	—	—	—	12.6	15.5	1.8 × 10^−1^	—	—	—	—
**GMS 4540**	16x	7.2–20.7	S3 (19.7)	13.7	14.9	5.4 × 10^−2^	S15 (7.7)	12.8	16	5.6 × 10^−2^
OW 1075	—	—	—	14.0	15.3	8.8 × 10^−2^	—	—	—	—

S#, Sensor label (e.g., S2); SZ, Saturated Zone; USZ, Unsaturated Zone.

The GMS 4027 (A) was installed down-gradient of a deep laboratory building that was completed in 2011 and extends to the bedrock. Data are available from January 2010 to May 2014 only, then the area around the GMS was built over. At GMS 4027 (distance to groundwater: approx. 11.5 to 12 m below ground surface (bgs)), GWT are only slightly affected by seasonal fluctuations in air temperature, but the comparatively high GWT indicates the influence of the deep building by heat radiation, most pronounced in the late winter months of the years 2011 and 2014, which coincides with comparatively cold winters and large heating demands [20]. In general, a strong positive trend of more than +0.2 K a^−1^ can be observed.

The GMS 4028 (B) was installed to capture the regional southwestern thermal regime. The groundwater table is at approx. 18 to 19.3 m bgs with two high GWH situations in the beginning of 2013 and summer 2016. At this depth, GWT is not expected to be affected by seasonal variations in air temperature, but GWT at 21.5 m bgs vary seasonally (1.2 K amplitude). The relatively high GWT measured in nearby OW 1305 of up to 17.4°C is potentially caused by the OW being installed in a building’s basement. Temperatures in the USZ (sensor at 18.5 m bgs) vary seasonally between 14.5°C and 16°C. In general, a slight positive trend can be observed for the GWT (+0.06 K a^−1^) and unsaturated zone temperatures (UZT; value is +0.08 K a^−1^). While having a comparatively balanced thermal regime over the years, an abrupt GWT rise can be observed since summer 2019.

The GMS 4029 (C; groundwater at 15.5 to 17.5 m bgs) was installed nearby an industrial well used for cooling (warm water injection); almost down-gradient but—due to construction restrictions—with a slight shift to the side of the expected heat plume. In general, the GWT in GMS 4029 are similar to that for GMS 4028, with, among others, only slight GWT fluctuations (approx. 1.7 K amplitude), and positive trends of +0.06 K a^−1^ for GWT and +0.1 K a^−1^ for UZT. In 2013, GWT were relatively cold, while a steady rise of GWT was recorded since summer 2017. Up to now, a direct influence of the up-gradient warm water injection is not directly recognizable—most likely, the spreading of the heat plume is less pronounced than expected (see local-scale models in [22]).

The GMS 4030 (D) was installed near the river Rhine. The groundwater table of two nearby OW is located at a depth of approx. 11 to 13 m bgs, with the influence of the river Rhine level and flood events clearly apparent. Seasonal variations of air temperature can be observed within the USZ (amplitude of 3 K), which also affects GWT from 2018 on. At depths of about 14 to 15 m bgs as well as 20 to 21 m bgs, respectively, stratification of slightly warmer groundwater is evident, indicating preferentially advective heat spreading in this layer. In general, a positive trend can be observed for the GWT (+0.04 K a^−1^) and USZ temperatures (+0.18 K a^−1^). Since summer 2014, a steady rise of GWT was observed.

The GMS 4538 (E) was installed near the river Birs to study the thermal interaction between this tributary river and the aquifer. The groundwater is very shallow (approx. 1 to 3.5 m bgs only), and the influence of the river Birs level and flood events is clearly apparent. Seasonal variations of air temperature can be traced within the near surface with a pronounced amplitude of 26.3 K for UZT (at 0.9 m bgs), while GWT (deeper sensors at 11.4 m bgs) vary seasonally, too, but clearly attenuated (1.8 K amplitude). The thermal regime in the SZ is comparatively balanced during the year. As before, a positive trend can be observed for the GWT (+0.02 K a^−1^). However, temperatures in the USZ show a negative trend (−0.17 K a^−1^). Because the temperature sensors of GMS 4538 are located near the surface, this GMS provides valuable insights into the USZ’s thermal regime and enables estimating the heat input into the subsurface via the atmosphere and sealed surfaces in this urban area. This information was used for the 3D-TH models.

The GMS 4539 (F) was installed down-gradient of a recently (2007–2016) developed urban area in Kleinbasel. The GWH varies between 7.5 to 11 m bgs and GWT varies seasonally to a mediocre extent (3.5 K amplitude) while USZ temperature fluctuates between 12.2°C and 19.4°C. As for GMS 4538, an attenuation of the temperature fluctuation is observed, but with a very strong positive trend for the GWT (+0.37 K a^−1^) and for UZT (+0.29 K a^−1^). This strong positive trend can also be observed in two nearby conventional OW (see previous section and table 1). From 2020 onward, the trend appears to cease, which may indicate that the initial thermal influence of the newly developed urban area has ceased and that a new thermal equilibrium has been reached. The increase in GWT can be an indication of heat input into the subsurface by the buildings during the first heating period in winter 2015/2016. Therefore, this GMS is suitable for the long-term documentation of thermal influences of the recently developed urban area.

Finally, the GMS 4540 (G) was installed at the inflow of an industrial area (mainly used by a pharmaceutical company) in Kleinbasel. The groundwater table is located at a depth of approx. 9.5 to 13 m bgs; even strong GWH of up to almost 3 m appear to have hardly any effect on the GWT (small amplitude of 1.2 K only). Contrary to this, USZ temperatures clearly mimic air temperature seasonality, with values between 12.8°C and 16.0°C. Since 2017, a positive trend can be observed (between approx. +0.05 and +0.1 K a^−1^).

### (c) Implementation of the 3D-TH models

**Figure 3.**
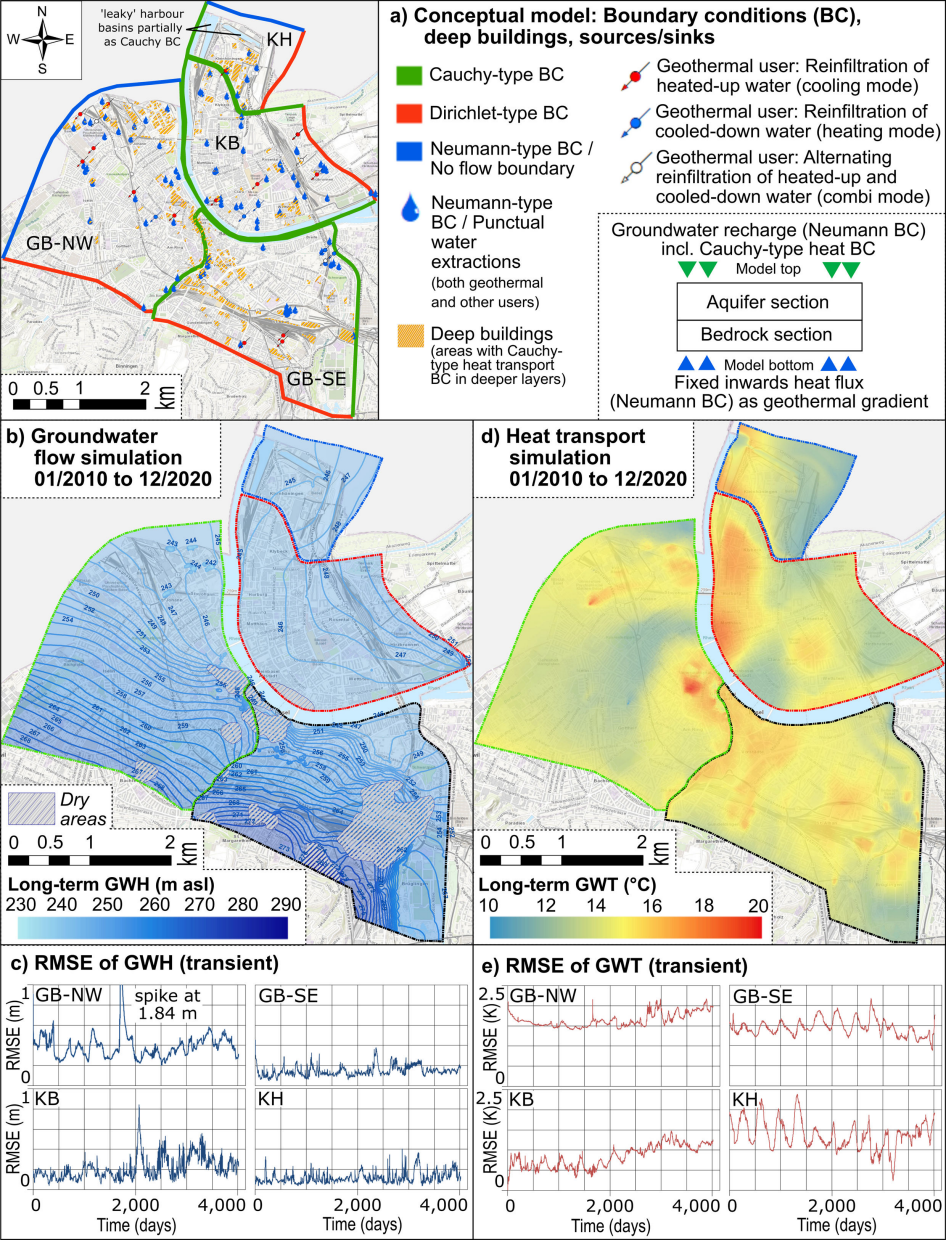
Schematic overview of the numerical model implementation for the four GWB (a) including the main BC. The simulated averaged GWH (b) and GWT (d) for the full simulation period are exemplarily shown. The development of RMSE over time is provided for both GWH (c) and GWT (e). Hereby, the calibration period ranges from 0 days to 2192 days; the validation period from 2192 to 4018 directly follows.

A mere interpolation between the GWT measured at OW and GMS would not account for the local-scale heterogeneity of the thermal impacts, especially not under transient conditions. Hence, combined groundwater flow and heat transport was simulated in all four GWB with FEFLOW^©^. In this process, the previous versions (latest version by [17]) were updated and overhauled. The conceptual setup, including boundary conditions (BC) and other model objects such as buildings and sinks/sources, is illustrated in figure 3a and briefly discussed in the following.

The model covers a total area of 22.4 km² (Kleinhüningen: 2.0 km^2^; Kleinbasel: 5.4 km^2^; Grossbasel Southeast: 6.7 km^2^; Grossbasel Northwest: 8.3 km^2^); the two major sections in the vertical, i.e. aquifer and bedrock, were subdivided into 12, 15, 18 and 23 layers, respectively. Most of these layers were assigned to the upper aquifer section and especially to the variably saturated part (i.e. the vadose zone). This required a mesh built of approx. 1.5 million three-dimensional elements (shape: triangular prisms; Kleinhüningen: 151 104 elements; Kleinbasel: 365 700 elements; Grossbasel Southeast: 441 144 elements; Grossbasel Northwest: 534 290 elements). The total simulation time for all four models was 4019 days (i.e. 11.0 years) covering the period from 1 January 2010 to 31 December 2020.

We employed the Richards equation (head-based) as implemented in FEFLOW^©^ for the flow simulation. As part of the TH approach, temperature dependencies for viscosity were considered in all runs to improve data continuity and interoperability with previous and local models (e.g. [22]) as well as to correctly address the effect of thermal sinks and sources. Especially in the very near vicinity of, e.g. geothermal wells, significantly increased temperatures may occur, eventually affecting hydraulic conductivities and solute transport parameters (e.g. [23]).

As depicted in figure 3a, the rivers (i.e. Rhine, Wiese, Birs and Birsig) were implemented as Cauchy-type (third type) BC, while the regional flow was defined by Dirichlet-type (first type) BC for both hydraulic and thermal simulation. Sinks and sources from (geothermal) wells were implemented as Neumann-type (second type) BC. Groundwater recharge and thermal interaction with the atmosphere were realized as Neumann-type BC and as Cauchy-type BC, respectively, in all areas without buildings. The buildings themselves were defined as de facto impermeable three-dimensional objects but with thermal relevance (also Cauchy-type). The geothermal gradient was mimicked by a constant heat flux assigned to the lowest layer of the bedrock. More GWB characteristics and implementation details can be found in the electronic supplementary material, tables S1–S3.

Calibration was performed by employing PEST [24]; the calibration period now comprises the years 2010 to 2015 (2192 days) and the validation period the years 2016 to 2020 (1827 days). While still employing the full TH modelling, i.e. both flow and heat transport are simulated in all runs of the four models, we used a step-wise approach for the calibration: first, the flow model with GWH as process variables of interest was calibrated and, subsequently, the heat transport model was optimized aiming to minimize temperature differences between observation and simulation. This stepwise method was used to keep the number of PEST-induced model iterations and, hence, total run times at a manageable level. Like the previous model version [17], the hydraulic calibration parameters (calibration step i) included the hydraulic conductivities and flow transfer rates (conductance) at the river boundaries. For the heat transport model (calibration step ii), the heat transfer rates of the topmost layer as well as selected heat transport parameters were considered as uncertain (longitudinal and transversal heat dispersivities, and porosities) and, therefore, were calibrated using the GWT. Where available and feasible, the multi-level GMS temperature in the unsaturated and saturated zones were involved as well. The coefficients were spatially interpolated by applying the pilot point method [25].

The results of the FEFLOW^©^ simulations are provided as long-term means (i.e. all time steps were merged) for both the GWH (figure 3b) and GWT (figure 3d). As an indicator for transient model performance, the root mean square error (RMSE) values are provided in figure 3c (GWH) and figure 3e (GWT). Electronic supplementary material, table S1 gives an overview of the RMSE averages (calibration and validation); the full time series are also digitally provided there. Here, values for the flow model part (GWH differences between simulation and observation) range from 0.10 m (GWB Kleinhüningen) to 0.39 m (GWB Grossbasel Northwest) for calibration and from 0.13 to 0.38 m (same GWB) for the validation period, while calibration- and validation-averaged RMSE values for the heat transport range from 0.63 K (GWB Kleinbasel) to 1.56 K (GWB Grossbasel Northwest) and from 1.08 to 1.75 K, respectively. Regarding the flow model, the GWB Grossbasel Northwest has comparably high discrepancies which can be attributed to a large groundwater flood event in the period of mid-2014 to mid-2015, where the groundwater hydraulics could not be reproduced for the northern region of this GWB. The heat transport model also requires mediocre improvements, but it is already a huge improvement to its prior state, especially for the GWB of Kleinbasel with a RMSE during calibration of approx. 1 K and less. Many of the observed SUHI effects could be reproduced. Discrepancies are addressed, among others, to (i) the unavoidable methodical simplifications and assumptions used in the GWB, such as uniform temperature time series for all buildings and for the industrial geothermal users (assuming that these users follow the regulations), (ii) the existing knowledge gap related to smaller (also geothermal) groundwater users (only the industrial wells are implemented), and (iii) the only indirect representation of the areal thermal impactors (district heating supply, sewer networks) as part of the calibrated heat exchange BC with the atmosphere.

## 4. Discussion

### (a) Conventional OW versus high-resolution multi-level GMS

The different monitoring networks, specifically the conventional OW as well as the multi-level GMS, provide information on the long-term, spatiotemporal evolution of GWT in Basel City. These data formed a robust base for the updated, high-resolution 3D-TH models. Both monitoring systems highlight the positive trend of GWT in all investigated areas over the monitoring period confirming transient SUHI development. Nevertheless, an important aspect for a correct evaluation here is the construction of monitoring systems that allow not only for the identification of approximate trends for larger regions, but also for the scientifically correct definition and quantification of thermal BC. However, conventional OW monitoring is often, e.g. by technical aspects, not intended to trace GWT in such detail, so that only rough assumptions on thermal BC are possible. Mostly, the spatial distribution of the OW, while being acceptable for hydraulics and groundwater sampling, is unsuitable for correctly reflecting the thermal heterogeneity in urban regions. To evaluate local effects, nearby and well-positioned on-site monitoring systems are necessary to distinguish between the thermal contributor itself (e.g. a specific building) and other, overlaying, more areal features, such as the natural contribution by elevated air temperatures. The specific location of a single OW may also not be suitable for recording GWT. For example, some conventional OW of Basel’s monitoring network are positioned in basements (e.g. OW 1305) due to limited space available; these OW are most likely biased by the building heating, leading to a positive offset of temperature values. The sewer networks, especially the main sewer lines, are also assumed to bias some OW (e.g. OW 703), where significantly elevated GWT were observed (much higher than adjacent OW). Finally, most conventional OW are employing single-sensor systems (i.e. one temperature sensor per well in a defined depth). Here, convective heat transport and vertical water mixing within the OW can falsify the monitored GWT value. Because the seven installed multi-level GMS in Basel City are embedded directly in the ground (i.e. not in an OW), they are excepted from these processes and, therefore, allow for way more detailed process studies and for defining BC for TH modelling of the investigated area.

Nevertheless, a conventional OW network is a crucial base requirement for adequately evaluating the current thermal state of the groundwater. It allows for monitoring hydraulics and GWT on an acceptable quality level and at acceptable costs. However, these systems should be checked regularly for early detection of measurement offset and data gaps due to malfunctions. Further (partially multi-depth) temperature monitoring can and should be used to infer temperatures at specific sites during specific process studies, e.g. near deep buildings, reinjection wells or near surface waters (river–groundwater interaction). While being very beneficial for a better understanding of SUHI, these systems are more cost-intensive. A compromise must be made here by complementing site-specific high-resolution monitoring approaches with resource-saving, conventional approaches.

### (b) Benefits of high-resolution TH models based on the GWB approach

The ongoing development of the urban underground including the construction of infrastructure (e.g. parking lots, tunnels, sewer networks) and increasing geothermal use has led to long-lasting hydraulic and even more impactful thermal changes in the urban aquifers. Since, for example, the heat flux into the subsurface caused by buildings depends on various transient parameters, such as groundwater flow velocity, distance of the basement to the groundwater table [20] or seasonally varying air and surface water temperatures, a mere interpolation between points with available GWT would not account for the variety of thermal users in-between these points and thus dampening sustainable management efforts. To close this methodical gap, the application of TH models is not only recommended (e.g. [26]) but essential. Compared to conventional groundwater simulations, modelling thermal effects at city scale is way less common, often due to data scarcity hindering an adequate calibration.

The setup and update of the four high-resolution TH models of Basel City’s GWB allowed for quantifying water flow, heat flux and energy budgets in the shallow subsurface for the entire period between 2010 and 2020 using well-defined hydrogeological and thermal BC. The use of GWB as management units [3] allowed for a clear BC definition and for assessing the thermal potential of subdomains within the city. This information about thermal potential can be integrated into groundwater and energy management strategies. The simulation of the whole area of Basel City as a monolithically defined model, although technically possible and maybe feasible for the previous short-term 3D-TH models (1 year data for calibration), was unfeasible for the six-plus-five-year model since the calibration effort for each of the models already exceeded multiple weeks.

The re-developed 3D-TH models are works-in-progress themselves. The implementation of future time series (OW and GMS) is expected to identify currently unknown and/or potentially underrepresented thermal impacts. For example, the sewer network is assumed to have a noticeable impact [2,5]. However, integrating such comparably filigree objects into the FE models is, from a technical point of view, complex; early-stage investigations for possible surrogate methods are ongoing [5]. The same applies to a full three-dimensional representation of the subsurface heterogeneity of the gravel aquifer with hydrofacies-dependent assignments of hydraulic and thermal parameters (e.g. [27,28]).

### (c) The importance of keeping a well-maintained data inventory

Conventional groundwater usage (drinking water, industrially used water) and groundwater-related energy industry strongly interact. Protective measures must be defined by authorities to reach the goals of sustainable management, balancing the needs of subsurface and groundwater use against groundwater preservation. Suitable monitoring and modelling tools must be selected and implemented to assess (i) the current thermal state, (ii) the thermal potential of the shallow subsurface, and (iii) the changes of the thermal state that may be introduced by the measures taken (e.g. reinjection of cold water into a GWB). The quantitative and qualitative assessments of (i) to (iii) require a well-maintained data inventory for each urban GWB including hydro(geo)logical and anthropogenic settings (mostly stationary character) and BC (mostly transient character), see also [3]. For this study, data from multiple authorities and companies were acquired—typically in a large variety of data formats—and transformed into numerically implementable forms. Unfortunately, due to the complexity of the thermal regimes, there is no generalized or standardized approach possible. However, defining a replicable standard procedure for a given location (here Basel City), i.e. setting up a long-term data inventory and following a good documentation, is an absolute necessity to avoid re-inventing the wheel for each model update.

This data inventory can further be used to set up future 3D-TH models for specific tasks, both on local and GWB scales. Here, one challenge not to be underestimated is to select adequate monitoring sites to understand thermal processes correctly. Pre-processing and local-scale tools to investigate these specific processes may include simplistic analytical models (typically one-dimensional), GIS-based approaches such as interpolation methods, local two-dimensional-TH or 3D-TH models (e.g. [22]), or a combination of them (this study).

## 5. Conclusions and future challenges

Previous works in Basel City already developed and applied high-resolution subsurface temperature monitoring concepts and 3D-TH modelling tools for selected scenarios. In the present study, we further developed, partially re-evaluated and adopted these tools and procedures for long-term assessments at the city scale, allowing for a sustainable management of subsurface resources such as groundwater quantity and heat fluxes. Specifically, we tested the applicability of tools and procedures with a more than decade-long dataset by (i) evaluating the GWT trend since 2010 in Basel City for the four GWB of Grossbasel Northwest, Grossbasel Southeast, Kleinbasel and Kleinhüningen; by (ii) evaluating high-resolution, multi-level temperature data in the vadose and in the groundwater-saturated zone at seven GMS locations highlighting both anthropogenic impacts as well as natural effects; and, finally, by (iii) thoroughly updating FEFLOW^©^-based 3D-TH models for those four GWB. These steps and tools in combination allow for formulating scenarios for the future use of energy for urban districts of Basel City. Although changes in air temperatures are contributing as well, the transient progress of SUHI, revealed by elevated GWT in Basel City, is a clear result of ongoing anthropogenic activities including surface sealing, thermal industrial-size groundwater users and the construction of deep buildings reaching or exceeding the groundwater table. This was shown by the depth-oriented datasets and could be mimicked to a large extent by the modelling results.

The updated 3D-TH models can and will be used in energetic planning and strategy development at the city scale to address multiple challenges related to a sustainable thermal management of the urban subsurface of Basel City. This includes (non-exhaustive list): the exploration of the energetic subsurface potential of urban districts that will be rebuilt or restructured, the assessment of possible thermal interferences of groundwater users (e.g. groundwater heat pumps), or the conduction of forecasts and feasibility studies during planning and construction periods of underground infrastructures (tunnels or deep buildings). Requirement-dependent analyses can flexibly be performed with the 3D-TH models, and different spatial variants of planned tunnel transects can be integrated. To cope with ongoing system and regime changes (variations of hydraulics and of thermal stressors), groundwater and energy management strategies and the measures introduced by authorities should be iteratively re-evaluated based on monitoring and simulation results.

## Data Availability

Where possible, metadata and relevant datasets are provided in the manuscript directly and extensive supplementary material, including two Excel files containing observed as well as simulated groundwater temperature data and groundwater heads. Due to privacy concerns, legal restrictions and third-party ownership, some specific data such as thermal user data may not be publicly accessible. Supplementary material is available online [29].
